# Seismic rehabilitation of steel buildings with semi-rigid connections under fifth generation of ETEF

**DOI:** 10.1038/s41598-026-47204-3

**Published:** 2026-04-05

**Authors:** Ali Majdi, Ataallah Sadeghi-Movahhed, Muhannad Riyadh Alasiri, Saiful Islam, Majid Movahedi Rad

**Affiliations:** 1 Department of Buildings and Construction Techniques Engineering, College of Engineering, Al-Mustaqbal University, Hillah, Babylon, 51001 Iraq; 2 Hillah, Iraq; 3 Civil Engineering Department, College of Engineering, King Khalid University, Abha, 61421 Saudi Arabia; 4 Department of Structural and Geotechnical Engineering, Széchenyi István University, Győr, 9026 Hungary

**Keywords:** Seismic isolation, Steel building, semi-rigid connections, Satchel connection, Rehabilitation, Engineering, Natural hazards, Solid Earth sciences

## Abstract

This study assesses the effectiveness of seismic base isolation using triple friction pendulum isolators (TFPIs) as a retrofitting strategy for a five-story steel building. This building is characterized by vulnerable Satchel connections, which suffered extensive brittle failures during the 1990 Manjil and 2003 Bam earthquakes in Iran. Four different experimentally calibrated Satchel connection configurations (S1 to S4) were modeled within a typical five-story building. These configurations varied in parameters such as rotational stiffness (780t.m/rad to 1380t.m/rad), angle lengths (15 cm to 20 cm), and beam sections (IPE180 and IPE220). For each of the four building models, all connections were uniformly assigned one of the four specimen types. The structural and non-structural performance of these models was evaluated using nonlinear endurance time (ET) analysis. The assessment covered seismic hazard levels corresponding to 475-year, 2475-year, and return periods beyond 2475 years. The results showed that implementing a base isolation system avoids the need for extensive strengthening of the existing connections. Moreover, by adding the isolators to the current connections, the structure’s performance can significantly surpass that of a brand-new, code-compliant design.

## Introduction

**Fig. 1:**
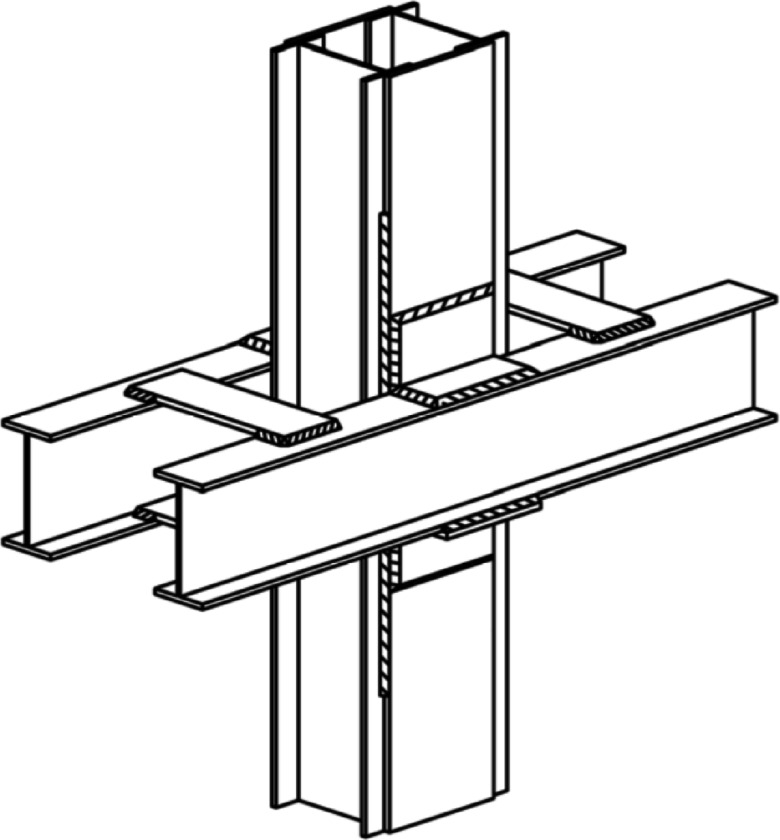
Schematic view of the Satchel connection^3^.

Beam to column connection is among the most critical design parameters in structures and it has been the subject of considerable research^1,2^. Deficiencies in these connections can cause structural damage during seismic events. This problem is especially common in older buildings. Serious concerns exist regarding the earthquake resilience of Iran’s older steel building stock. Historical earthquake damage, notably during the Manjil (1990) and Bam (2003) earthquakes, has proven that these buildings are prone to significant collapse and damage. The fact that vital infrastructure, such as hospitals and educational institutions, is included in this vulnerable group makes their potential failure a pressing issue, as these facilities must remain functional after a seismic event^3^. One of the most widely used connections in older Iranian steel buildings is the semi-rigid Satchel connection, locally known as Satchel. The semi-rigid Satchel connection is a simple and cost-effective method. It uses twin continuous beams that span over several columns and are structurally connected to the column sides using angle Sect^4^. Figure 1 shows a schematic view of the Satchel connection. Arbabi^5^ conducted a nonlinear finite element analysis to understand the behavior of the Satchel connection under monotonic and cyclic loads. The results showed that when properly designed, Satchel connections can demonstrate high stiffness in the elastic range. However, comparing the hysteresis loop to standard connections used in earthquake regions shows that Satchel connections may absorb less energy and are therefore less efficient at dissipating earthquake energy. Daryan and Bahrampoor^6^ investigated the fire behavior of Satchel connections through four elevated-temperature experiments and 3D finite-element (FE) modeling. The results confirm that Satchel connections cannot withstand temperatures exceeding 700 °C when using standard steel, which validates the FE model for use in fire design. Tehranizadeh^4^ examined the torsional dynamic response of Satchel connections via forced vibration tests on a half-scale four-story model. The results showed that connection stiffness significantly influenced torsional frequencies and eccentricity, so that higher stiffness reduced torsional coupling. Mostafaei and Mazroi^7^ evaluated the seismic performance of Satchel connections by conducting cyclic lateral loading tests on six mid-span specimens. The results indicated that reinforcing the Satchel connections, specifically through the use of triangular stiffeners, beam web stiffeners, batten plates, and plates on the beam’s top and bottom, was highly effective in increasing both moment capacity and rigidity. Shafei et al.^3^ proposed a method for improving the seismic performance of the Satchel connection using two vertical R-plates. The improved connection prevents weak-column/strong-beam failure by promoting plastic hinges in the beams. Tehranizadeh^8^ studied the impact of combining ADAS (Added Damping and Stiffness) dampers and chevron bracing on steel buildings featuring Satchel connections. The experiments showed that adding chevron and ADAS elements increased the frame’s first-mode frequency. Mirghaderi and Dehghani Renani^9^ developed two innovative Satchel connection details to upgrade their behavior from semi-rigid to fully rigid. The first uses two vertical plates welded to column flange edges and channel beam flanges for connection. The second employs four trapezoidal vertical plates welded to the column and I-beam flanges. Cyclic tests on all specimens per detail showed that all connections achieved an interstory drift of more than 0.08 rad without strength loss. Moreover, the results confirmed that the flexural behavior of the beam ends controlled the structural ductility. Hosseini Hashemi and Hassanzadeh^10^ analyzed a steel frame structure from the Bam earthquake, equipped with X-bracing, infill panels in both directions, and Satchel connections. Their findings indicate that the FEMA-356^11^ guidelines offer a reasonably accurate assessment of the building’s seismic performance.

As can be seen in previous research, structures with Satchel connections suffered widespread damage during major earthquakes such as Manjil (1990) and Bam (2003), primarily due to premature weld failure at the beam-column connections^3^. This vulnerability has established a clear need for effective retrofitting methods for existing steel buildings using this connection type. Therefore, researchers have proposed and investigated various retrofitting techniques aimed at strengthening these critical connections. This study introduces a novel and strategic shift in the retrofitting philosophy for such structures. Rather than focusing solely on strengthening the inherently vulnerable connection, we propose the application of a seismic isolation system at the base of the structure. Seismic isolators are proven, reliable systems that decouple the superstructure from ground motion, thereby reducing the seismic forces and deformation demands transmitted to the building, including its critical connections. The core innovation of this research lies in evaluating the effectiveness of this retrofitting strategy specifically for buildings with Satchel connections. It is investigated whether base isolation can sufficiently mitigate the seismic demands to prevent the premature brittle failure characteristic of these connections, thereby enhancing overall structural performance and safety. For this purpose, a steel structure with various Satchel connection specifications has been evaluated. Nonlinear Endurance Time analysis has been used for the evaluation of the structure’s performance under earthquakes with return periods of 475 years and 2475 years, as well as under earthquakes with return periods exceeding 2475 years.

### Numerical models

**Fig. 2:**
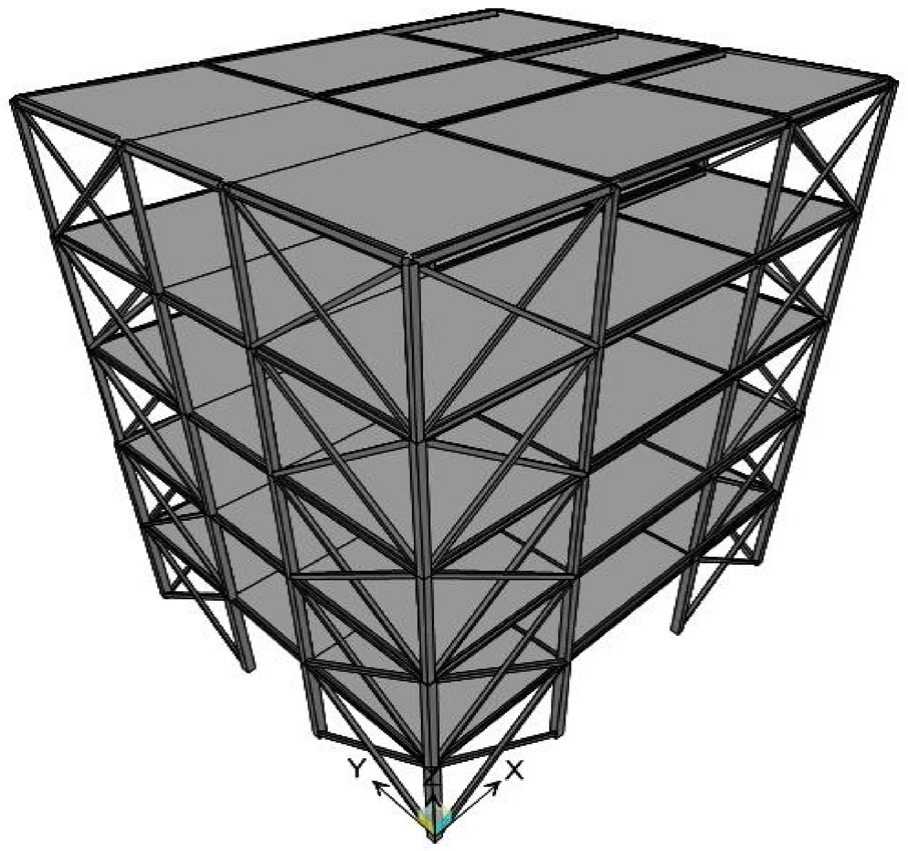
The 3D view of steel building with Satchel connections.

**Fig. 3:**
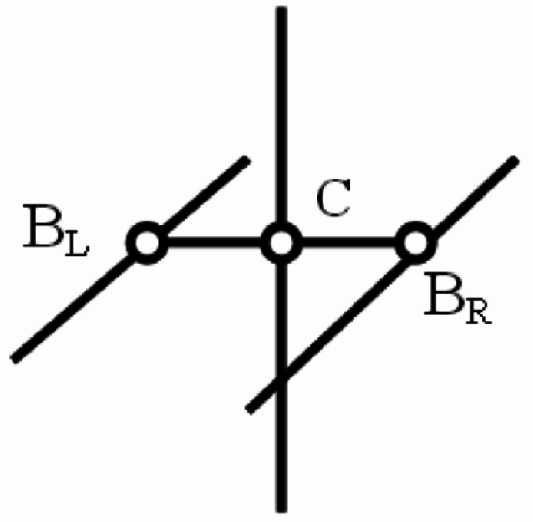
Modeling of the Satchel connections^14^.

In this study, a five-story steel building with Satchel connections is modeled (Fig. 2). The story height is 3.2 m. The frame consists of three bays in both the x and y directions. The length of the left and right bays in both directions is 4 m. Moreover, the length of the middle bay in the x and y directions is 5 m and 3 m, respectively. The dead and live loads for the floor are 6.3 kN/m^2^ and 2 kN/m^2^, which are reduced to 5.9 kN/m^2^ and 1.5 kN/m^2^ on the roof. The yielding stress, the ultimate stress, and the modulus of elasticity of the steel are${F_y}=23.5\;{\mathrm{kN/c}}{{\mathrm{m}}^{\mathrm{2}}}$, $\:{F}_{u}=36.28\hspace{0.33em}\mathrm{kN/c}{\mathrm{m}}^{2}$, and $\:{E}_{s}=20594\hspace{0.33em}\mathrm{kN/c}{\mathrm{m}}^{2}$, respectively. Floors and the roof are modeled as rigid diaphragms. Four experimentally derived Satchel connection types are considered in the model, with each type being analyzed individually. In other words, all structural connections are considered and evaluated separately based on each sample: once on S1, once on S2, once on S3, and once on S4. The details of these connections are presented in Table 1 based on the experimental work^12^. As shown in the table, the beam type and the cross-sectional dimensions of the top and bottom angles are similar in specimens S1 and S2. The only differing parameter is the angle length, which results in the differing values for the rotational stiffness of the connection and the ultimate flexural capacity between these two specimens. The same principle applies to specimens S3 and S4. Furthermore, the dimensions provided for the angles refer to the leg thickness and depth. For example, a designation of 80 × 80 × 8 mm indicates an angle with a leg thickness of 8 mm and a leg depth of 80 mm. The natural periods of the building, determined by modal analysis for connection types S1, S2, S3, and S4, are 0.5042s, 0.5044s, 0.5055s, and 0.5056s, respectively. The Satchel connection is modeled in SAP2000^13^ using two torsion bars connected to the column from both sides (Fig. 3)^14^. The displacements of the three nodes (BR, BL, and C) in three directions are interconnected using the constraint block available in the SAP2000^13^. The diameter of the torsion bar is calculated using Formula 1^14^.

1 $$\:d=\sqrt[4]{\frac{32kL}{\pi\:G}}$$

where $$\:k$$ is the torsional stiffness of the connection ($$\:\raisebox{1ex}{$kg.cm$}\!\left/\:\!\raisebox{-1ex}{$rad$}\right.$$); $$\:L$$ is the torsion bar length (cm) and $$\:G$$ is the shear modulus ($$\:\raisebox{1ex}{$kg$}\!\left/\:\!\raisebox{-1ex}{${cm}^{2}$}\right.$$).

The soil is considered type C. The spectral response acceleration parameters are S_DS_ = 0.825 g and S_D1_ = 0.614 g.

**Fig. 4:**
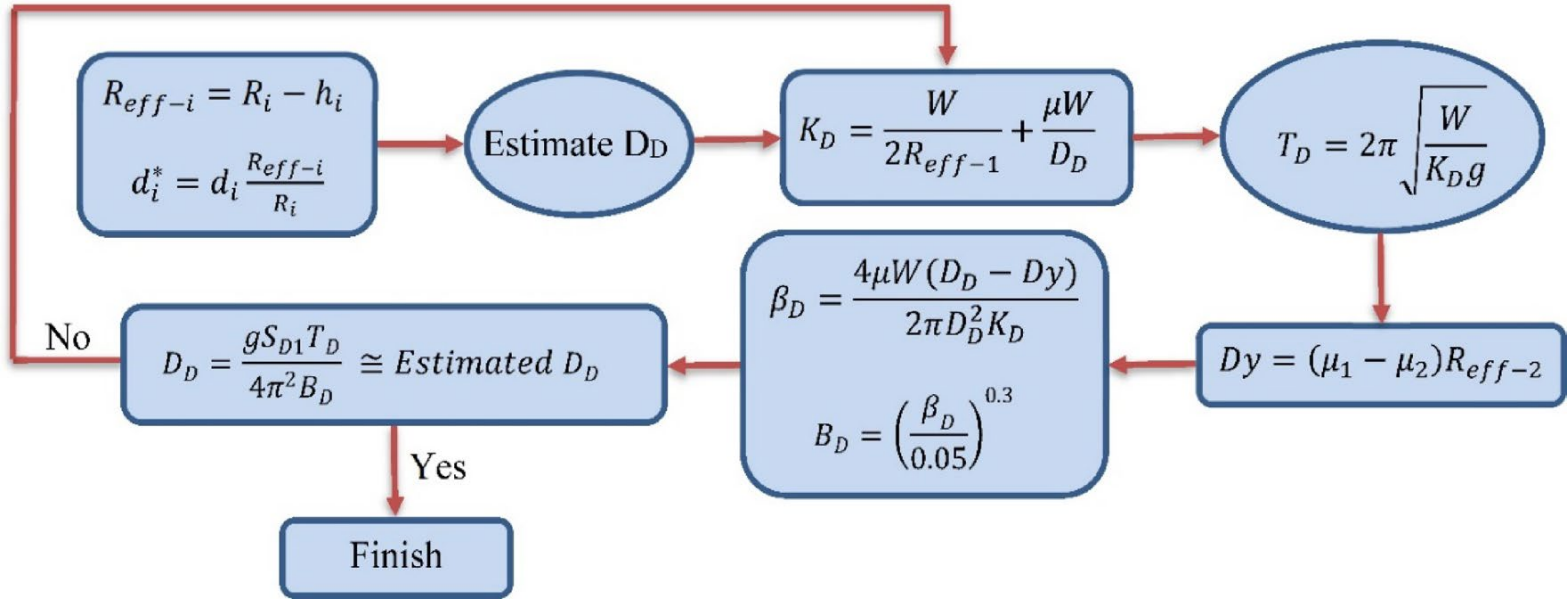
Isolator design steps^24^.

The Triple Friction Pendulum Isolator (TFPI) is an improved version of the single-friction pendulum isolator. Previous studies have comprehensively investigated the TFPI performance^15–19^. The design and modeling of Triple Friction Pendulum Isolators (TFPIs) are performed using the Triple Pendulum Isolator link element in SAP2000 [13]. The design procedure, outlined by Constantinou et al.^20^ (Fig. 4), is an iterative process. It involves first estimating the isolator displacement ($\:{D}_{D}$), then calculating the system’s dynamic properties, including effective stiffness ($\:{K}_{D}$), period ($\:{T}_{D}$), damping ratio ($\:{\beta\:}_{D}$), and damping reduction factor ($\:{B}_{D}$). Finally, checking the calculated displacement against the initial estimate. This cycle repeats until the values converge. Furthermore, the model assumes zero structural damping to comply with Sarlis and Constantinou’s^21^ recommendation for preventing damping leakage. Table 2 summarizes the relevant isolator properties. The friction coefficients for the isolator’s external ($\:{\mu\:}_{1}={\mu\:}_{4}$) and internal surfaces ($\:{\mu\:}_{2}={\mu\:}_{3}$) are 0.09 and 0.05, respectively. Furthermore, the isolator’s period, which has a critical effect on the isolated building’s performance^22,23^, is 1.9s.

**Table 1:** The design parameters of Satchel connections.

Specimen	Beam section	Top angle (mm)	Bottom angle (mm)	Angle length (cm)	Rotational stiffness of connection (t.m/ rad)	Ultimate flexural capacity (t.m)
S1	IPE180	80 × 80 × 8	100 × 100 × 10	15	780	4.9
S2	IPE180	80 × 80 × 8	100 × 100 × 10	20	1229	7.95
S3	IPE220	100 × 100 × 10	120 × 120 × 12	15	1181	6.75
S4	IPE220	100 × 100 × 10	120 × 120 × 12	20	1380	8.75

In Fig. 4; Table 2, $\:{R}_{eff-i}$ is the isolator effective radius; $\:{d}_{\mathrm{i}}$ and $\:{d}_{i}^{\mathrm{*}}$ are the nominal and actual displacement capacity, respectively; W is the effective seismic weight of the building; $\:\mu\:$ is the isolator total equivalent friction coefficient; g is the gravity acceleration; $\:Dy$ is the isolator yield displacement.

**Table 2:** Properties of the TFPI.

$\:{\mu\:}_{1}={\mu\:}_{4}$	$\:{\mu\:}_{2}={\mu\:}_{3}$	$\:{R}_{eff1}={R}_{eff4}$ (cm)	$\:{R}_{eff2}={R}_{eff3}$ (cm)	$\:{d}_{1}={d}_{4}$ (cm)	$\:{d}_{2}={d}_{3}$ (cm)	$\:{T}_{D}$ (sec)
0.09	0.05	213	33	40	1.5	1.9

### Analysis method

**Fig. 5:**
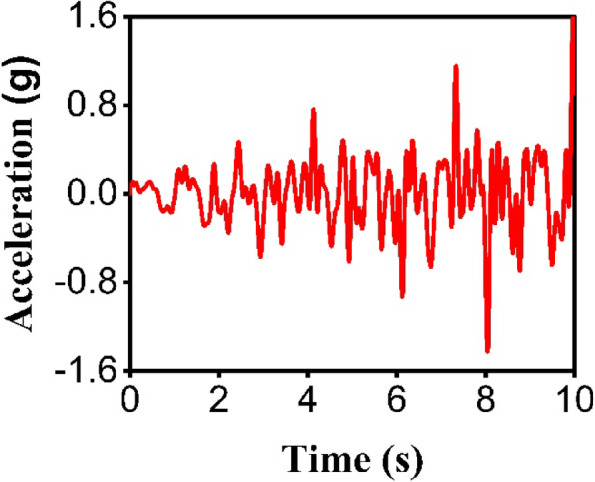
The ETA40kdx.

 The endurance time (ET) method, a rapid dynamic analysis technique still in development for various structures^25,26^, is used for structural evaluation. It applies a synthetic accelerogram that integrates features from multiple real earthquake records, optimized into a single function^27^. These are called endurance time excitation functions (ETEFs), with intensity escalating over time. Here, ETA40kdx (Fig. 5) is applied, created via the fifth-generation approach from 22 FEMA-P695 far-field records^28^. Therefore, responses under ETA40kdx match the average from those 22 records, greatly lowering computational demands. The development of the fifth generation of ETEF records relies on the simultaneous consideration of multiple dynamic response indicators. These crucial metrics include the spectral acceleration, the nonlinear spectral displacement, and the energy dissipated through hysteretic behavior. The requirement for absorbed hysteretic energy is particularly important as it serves as a direct proxy for evaluating the extent of cumulative structural damage. A specific computational procedure is used wherein the acceleration spectra are defined by an ascending linear function. Following this initial specification, the equivalent nonlinear displacement spectra and the hysteretic energy functions are then analytically calculated. The entire process is governed by a mathematical objective function (designated as Eq. 1)^29^.

1 $$\:{F}_{ETEF}\left({a}_{g}\right)=\underset{0}{\overset{{T}_{max}}{\int\:}}\underset{0}{\overset{{t}_{max}}{\int\:}}\left\{{\left[{S}_{a}\left(T,t\right)-{S}_{aC}\left(T,t\right)\right]}^{2}\right\}{d}_{t}{d}_{T}+\underset{1}{\overset{{\mu\:}_{max}}{\int\:}}\underset{0}{\overset{{T}_{max}}{\int\:}}\underset{0}{\overset{{t}_{max}}{\int\:}}\left\{\genfrac{}{}{0pt}{}{{a}_{{u}_{m}}\left[{u}_{m}\left(t,T,\mu\:\right)-{u}_{mC}\left(t,T,\mu\:\right)\right]+}{{a}_{{E}_{H}}\left[{E}_{H}\left(t,T,\mu\:\right)-{E}_{HC}\left(t,T,\mu\:\right)\right]}\right\}{d}_{t}{d}_{T}{d}_{\mu\:}$$

where *S*_*a*_(*t*, *T*) is the ETEF acceleration spectra at time $\:t$ and in the period $\:T$; *S*_*aC*_(*t*,*T*) is the ETEF target acceleration spectra; *u*_*m*_(*t*,*T*,*µ*) is the nonlinear SDOF displacement demand; *u*_*mC*_(*t*,*T*,*µ*) is the target nonlinear displacement; *E*_*H*_(*t*,*T*,*µ*) is the nonlinear SDOF hysteretic energy demand under ETEF; *E*_*HC*_(*t*,*T*,*µ*) is the target hysteretic energy demand.

## Results and discussion

This section evaluates the effectiveness of a Base Isolator in protecting both structural and non-structural elements of a steel frame equipped with varying Satchel connection properties. The evaluation is conducted using ET curves under both Expected and Unexpected Seismic Hazards. The analysis also includes an assessment of the structure’s base shear response. The structure’s performance is analyzed by comparing responses with and without the Base Isolator. The analysis employs seismic hazard levels associated with 475-year and 2475-year return period earthquakes. In cases where region-specific risk spectra are absent for return periods greater than 2,475 years, the 2,475-year spectrum is scaled up by factors between 1.5 and 2.0.

### Structural elements

According to ASCE 41 − 06^30^, the seismic performance of braced steel frames is classified into three levels based on the interstory drift ratio (IDR) and residual interstory drift ratio (RIDR): Immediate Occupancy (IO), Life Safety (LS), and Collapse Prevention (CP). The corresponding IDR limits for these levels are 0.5%, 1.5%, and 2%, respectively. Moreover, the RIDR limits are negligible, 0.5%, and 2%, respectively.

**Fig. 6:**
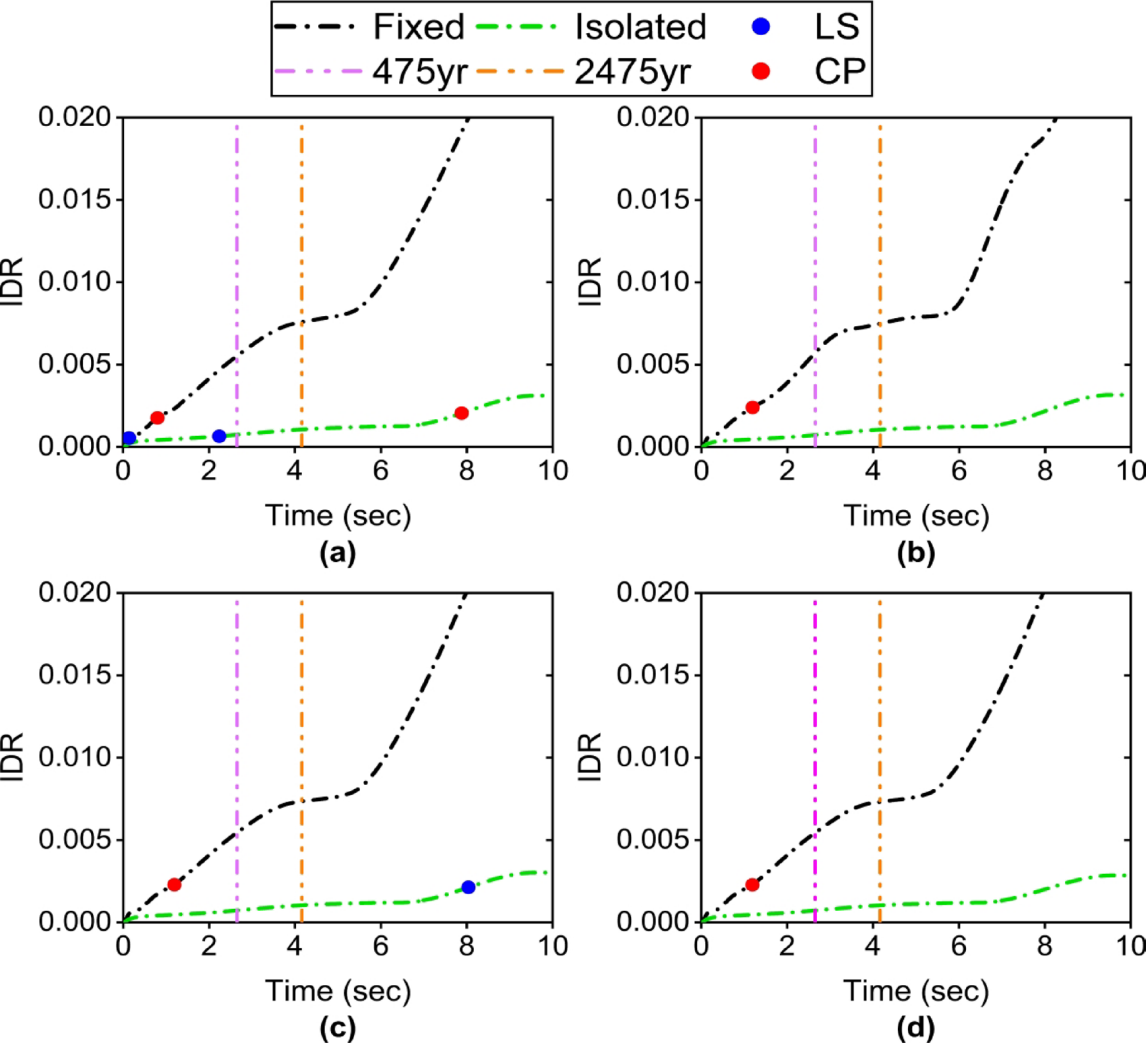
ET curve of IDR: (**a**) S1 specimen; (**b**) S2 specimen; (**c**) S3 specimen; (**d**) S4 specimen.

**Fig. 7:**
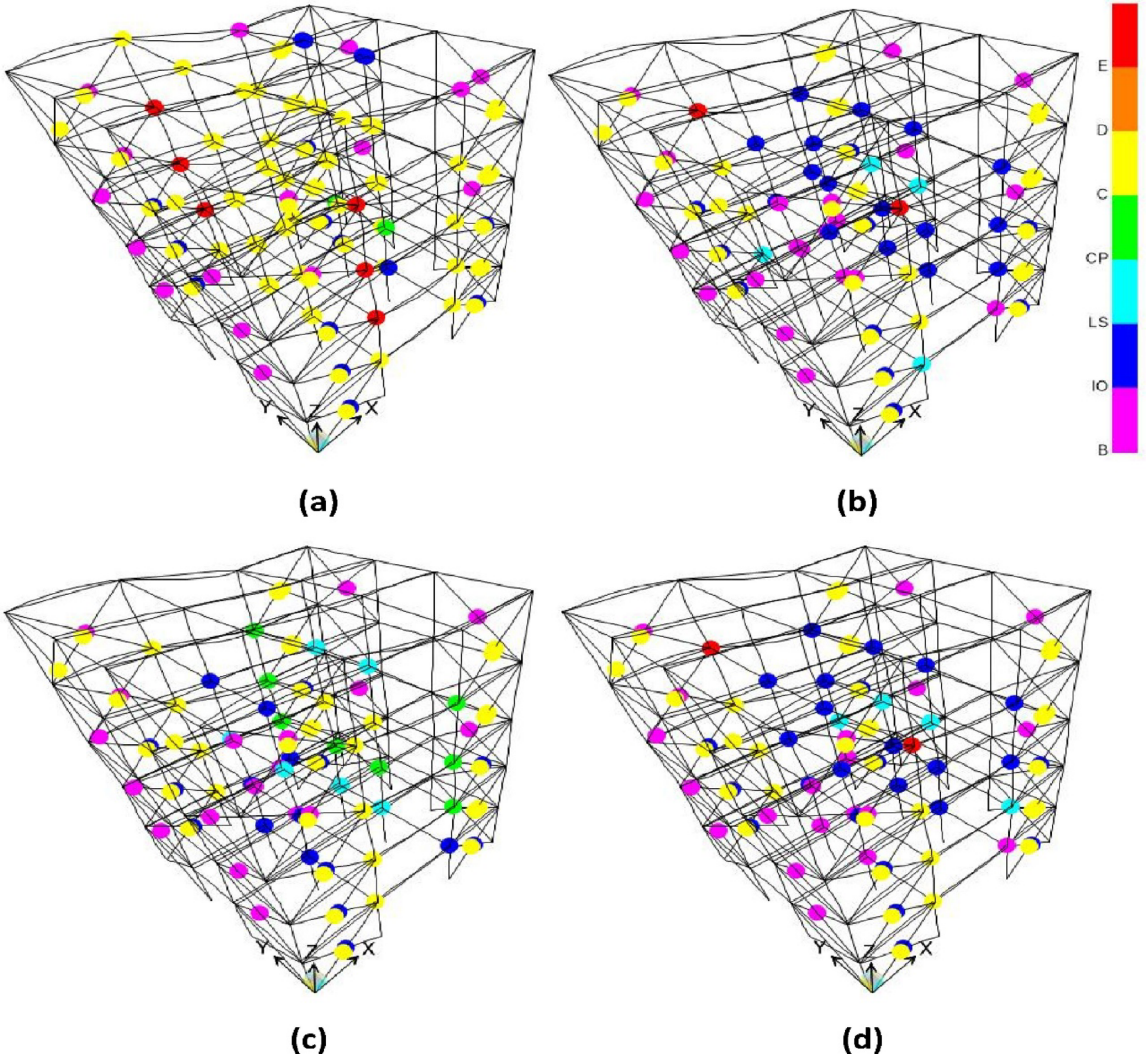
Hinges condition under 475 year seismic level: (**a**) S1 specimen; (**b**) S2 specimen; (**c**) S3 specimen; (**d**) S4 specimen.

The curves in Fig. 6 illustrate the IDR ET curve of four Satchel connection specimens (S1 to S4) under two conditions: isolated and non-isolated. In all four specimens, the behavior of the fixed-base structure is extremely brittle and susceptible to damage. The fixed-base structure in specimens S1 and S2 reaches IDRs of 0.0055 and 0.0057 under the 475-year return period earthquake level, respectively, placing them at the LS performance level. However, the state of the formed plastic hinges (Fig. 7a and b) indicates a performance level closer to CP (red point). Similarly, specimens S3 and S4 also reach IDRs of 0.0055 and 0.0054, respectively, achieving the same performance level as the previous two connection types (Fig. 7b and c). The fixed-base structure in all specimens reaches an IDR of approximately 0.0074 under the 2475-year return period earthquake level, and a significant number of elements enter the collapse failure region.

In contrast, the isolated structure exhibits significantly improved behavior. The maximum IDR in all four isolated specimens is less than 0.0008 under the 475-year return period earthquake level, representing a reduction by a factor of approximately 7 compared to the fixed-base case. For example, the IDR of S1 decreases from 0.0055 to 0.00074. Moreover, the isolated configuration reduced the IDR by a factor of 5.5, limiting it to approximately 0.001 under the 2475-year return period earthquake level. Regarding plastic hinge formation, only specimen S1 enters the LS range (blue point) at a seismic level below the 475-year level and remains at this level even beyond the 2475-year level. However, no plastic hinges form in the other specimens, even at seismic levels exceeding 2475 years.

The effect of increasing the stiffness of the Satchel connection from S1 to S2 (achieved by increasing the angle length from 15 cm to 20 cm) in the fixed-base case does not significantly affect the IDR but does influence the nonlinear hinge behavior. By comparing the hinge conditions in Fig. 7, it can be observed that fewer structural elements in specimen S2 enter the collapse stage than in S1. This reduction is attributed to the enhanced participation of the connections in energy dissipation at stories. Increasing the beam size from IPE180 to IPE220 and using larger angles from S2 to S3 significantly reduced the number and condition of plastic hinges. Conversely, this increase in stiffness is largely ineffective in the isolated case, even at seismic levels exceeding 2475 years, because the isolator dissipates most of the seismic energy and the superstructure develops approximately no plastic hinges.

**Fig. 8:**
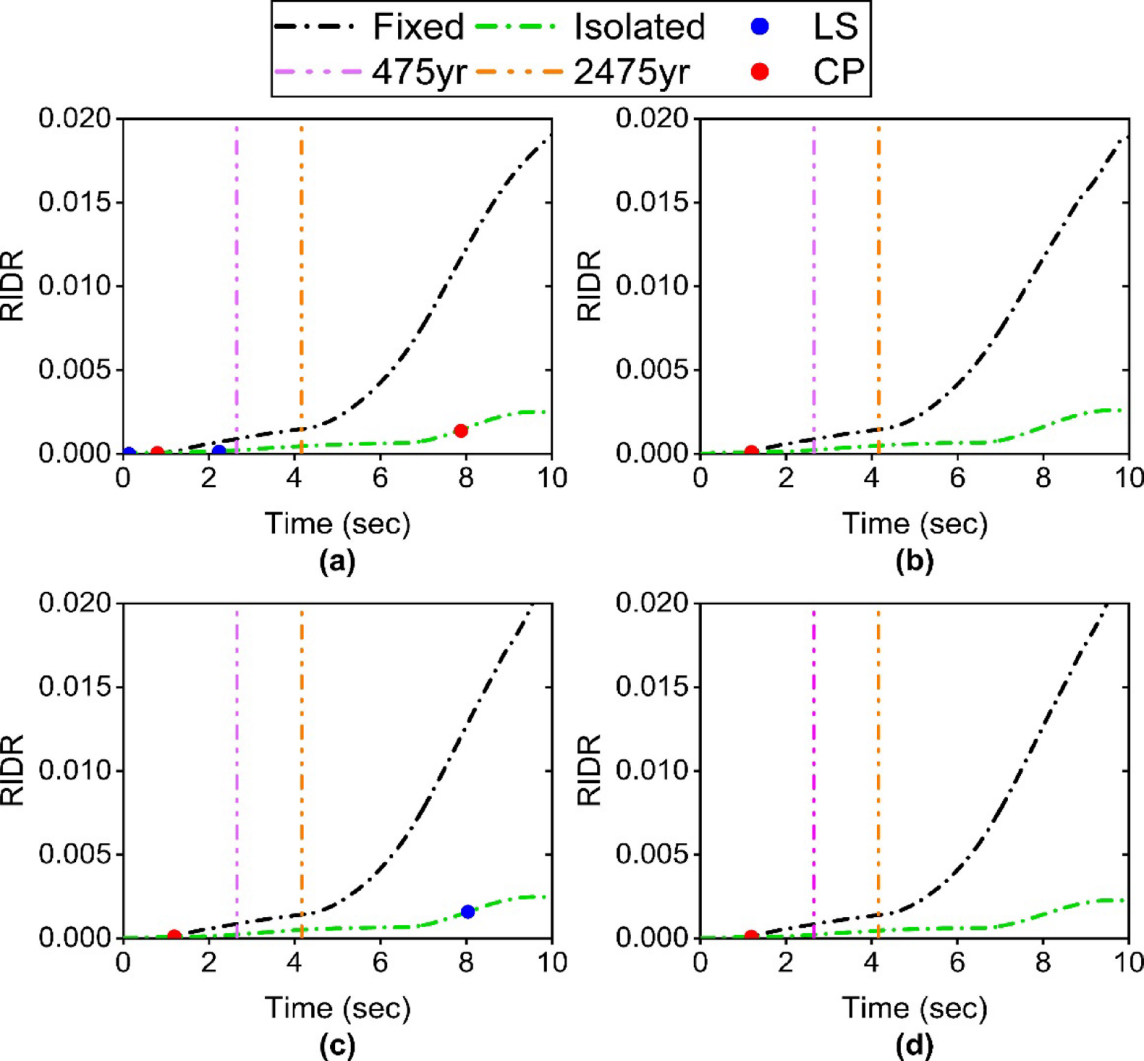
ET curve of RIDR: (**a**) S1 specimen; (**b**) S2 specimen; (**c**) S3 specimen; (**d**) S4 specimen.

Figure 8 shows the RIDR ET curves for the structure with different Satchel connections. The trend of RIDR variation in the base-isolated structure, compared to the fixed-base structure, is similar to the trend of IDR variations. Both the fixed-base and base-isolated structures with different Satchel connections met the IO performance level under earthquakes with return periods of 475 and 2475 years, according to the code classification. However, it should be noted that the plastic hinge state indicates the fixed-base structure entered the CP performance level (red point) before reaching the 475-year intensity level. Therefore, the RIDR results for the fixed-base structure should be evaluated based on the CP performance level. In contrast, the base-isolated structure maintains the IO performance level in terms of both plastic hinge formation and code-based criteria.

Another noteworthy point is that the RIDR for the fixed-base structure shows an increasing trend, while the isolator has kept the RIDR within a specific range, even for intensities beyond the 2475-year return period. This demonstrates the isolator’s high capability to preserve the multi-story superstructure’s performance as a single-degree-of-freedom system, a finding consistent with previous research on structures with other types of lateral force-resisting systems.

The color legend in the Fig. 7 indicates different hinge states: B represents the effective yield point; IO, LS, and CP correspond to the immediate occupancy, life safety, and collapse prevention performance levels, respectively; C marks the beginning of significant strength degradation; D indicates the residual strength phase; and E denotes that the element’s strength is essentially zero.

### Non-structural acceleration-sensitive elements

Absolute floor acceleration is the most important parameter for assessing damage to acceleration-sensitive non-structural components (such as medical equipment, electrical installations, partition walls, and false ceilings). For acceleration-sensitive non-structural components under a high code, HAZUS4-2^31^ specifies four damage states: Slight (0.3–0.6%), Moderate (0.6–1.2%), Extensive (1.2–2.4%), and Complete (≥ 2.4%).

**Fig. 9:**
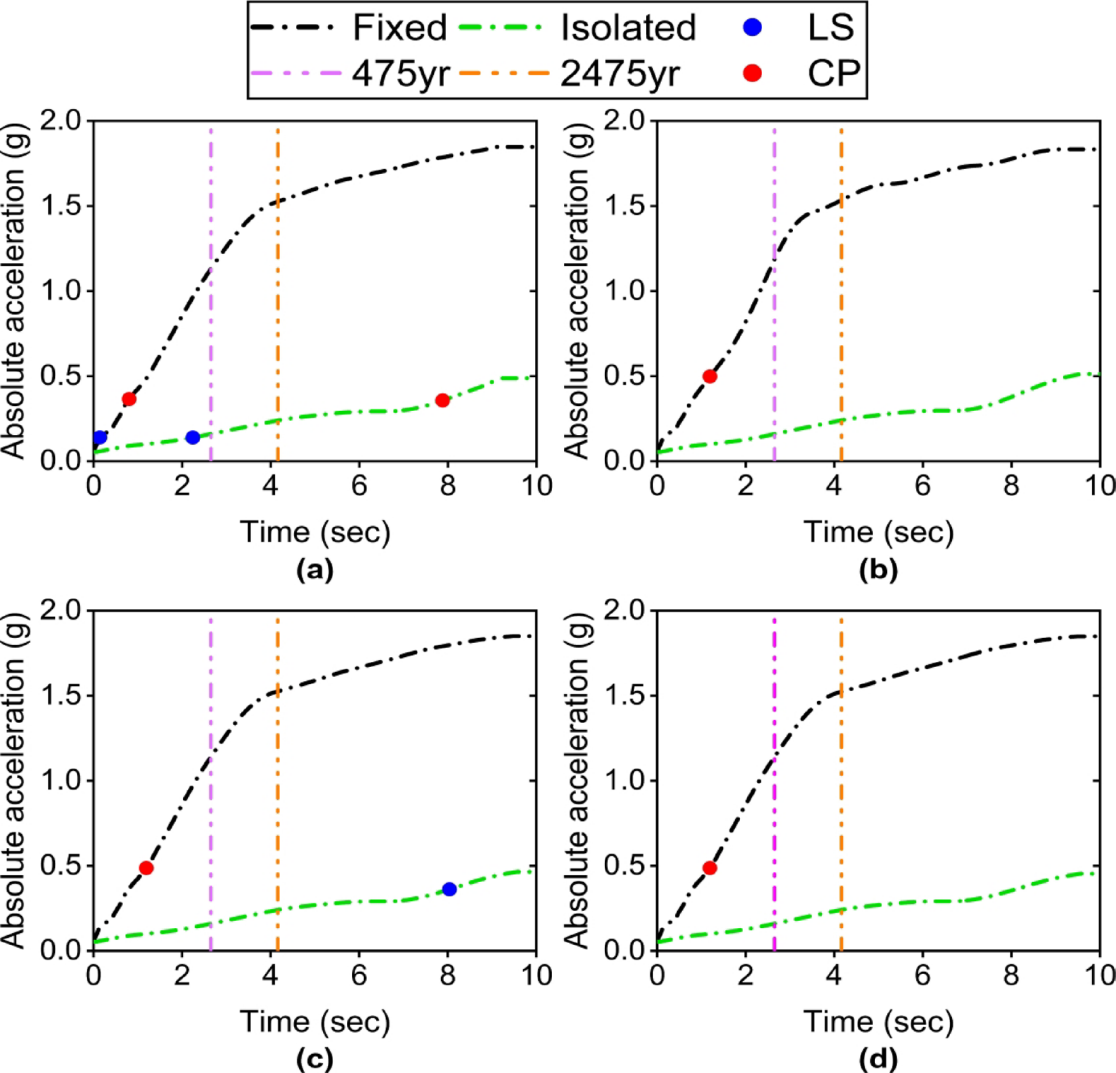
ET curve of acceleration: (**a**) S1 specimen; (**b**) S2 specimen; (**c**) S3 specimen; (**d**) S4 specimen.

The acceleration response is very severe in the fixed-base condition. Under the 475-year return period level, the peak acceleration in specimens S1 to S4 reaches approximately 1.13 g, 1.16 g, 1.14 g, and 1.15 g, respectively (Fig. 9). These values fall within the extensive damage range. Moreover, under the 2475-year return period level, the peak acceleration in specimens S1 to S4 reaches approximately 1.52 g, 1.53 g, 1.53 g, and 1.54 g, respectively, still remaining within the extensive non-structural damage range. The use of an isolator induces a remarkable reduction in acceleration. The peak acceleration in all specimens decreases significantly to 0.16 g (an approximate 7-fold reduction) under the 475-year return period level. Furthermore, the maximum acceleration in the isolated case is 0.24 g (an approximate 6.3-fold reduction) under the 2475-year return period level, which falls within the no damage range. Even at the most severe point (at the 10-second mark), the peak acceleration reaches 0.45 g to 0.51 g, corresponding to slight damage in the worst-case scenario. This clearly demonstrates that the isolator successfully downgrades non-structural damage from extensive to slight.

In the fixed-base case, increasing the connection stiffness leads to only a minor enhancement in acceleration. This is because stiffer connections slightly reduce the structure’s natural frequency. However, in the isolated case, the slight increase in acceleration due to stiffer connections is entirely negligible and can be disregarded. Furthermore, changing the beam section and the angles has little effect, as the accelerations in both isolated and non-isolated cases remain nearly identical.

### Base shear

**Fig. 10:**
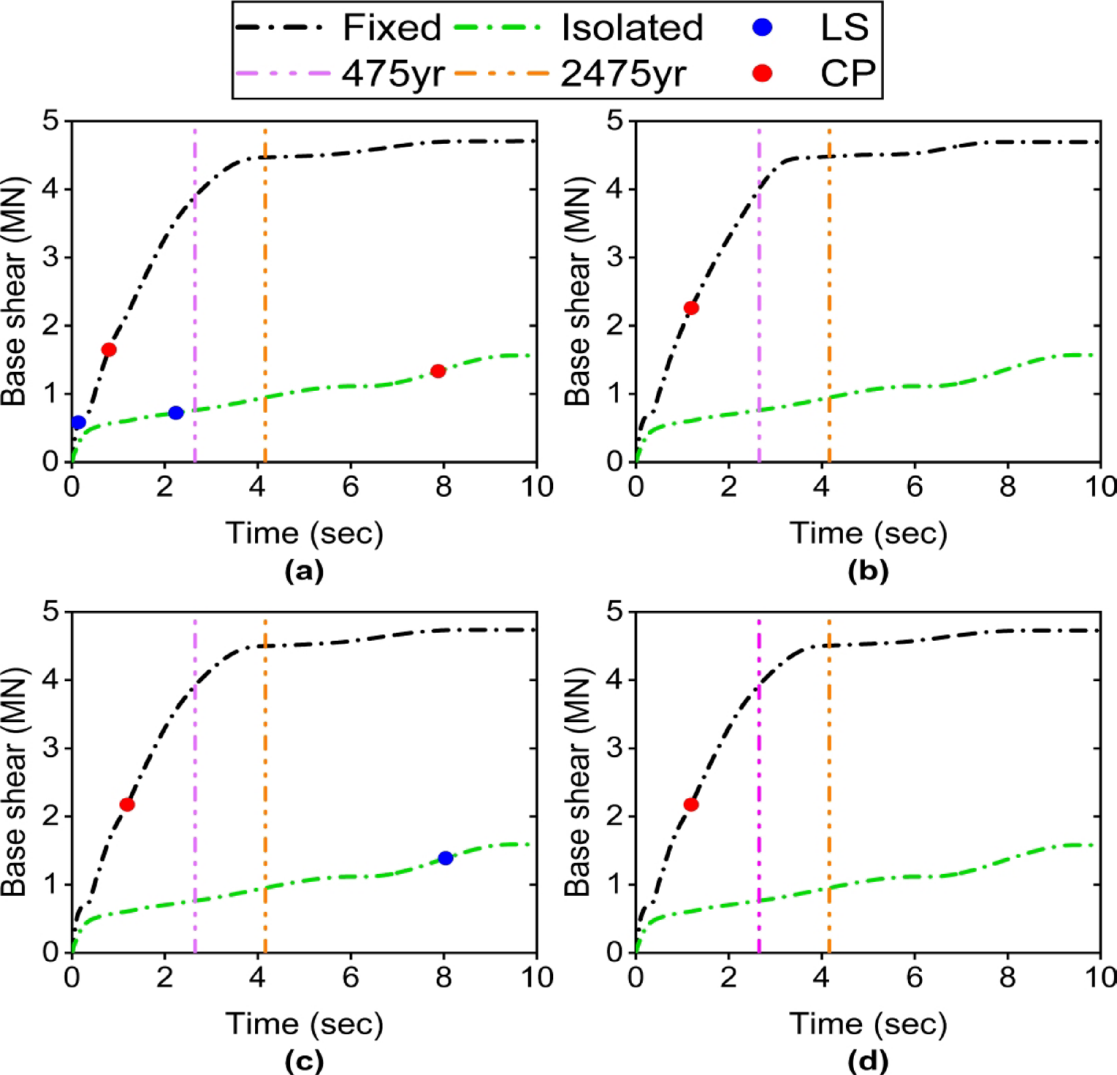
ET curve of base shear: (a) S1 specimen; (b) S2 specimen; (c) S3 specimen; (d) S4 specimen.

In the fixed-base case, the base shear curve rises steeply and reaches its maximum value (approximately 4.4MN to 4.6MN) within the intensity range corresponding to the 475-year to 2475-year return periods (Fig. 10). It then slightly decreases due to the formation of extensive plastic hinges. This value is nearly identical across all four specimens. This indicates that the increased stiffness of the Satchel connection has little effect on the maximum base shear, as the yielding of beams and columns becomes the governing factor under high force levels.

In the isolated case, the base shear is significantly reduced and the curve is much more gradual. Under the 475-year return period level, the base shear decreases from approximately 3.9 MN in the fixed-base case to 0.76 MN in the isolated case (a 5-fold reduction). Moreover, under the 2475-year return period level, the fixed-base shear is about 4.5 MN, whereas in the isolated case it is about 0.95 MN (a reduction of approximately 4.7 times). Even at the most severe intensity level, the isolated base shear reaches a maximum of only about 1.58 MN, which is less than 33% of the fixed-base case. Increasing the connection stiffness from S1 to S2 or using larger beam sections has almost no effect in the isolated case and the base shear remains nearly constant.

### Dissipated energy and force-displacement response of TFPI

**Fig. 11:**
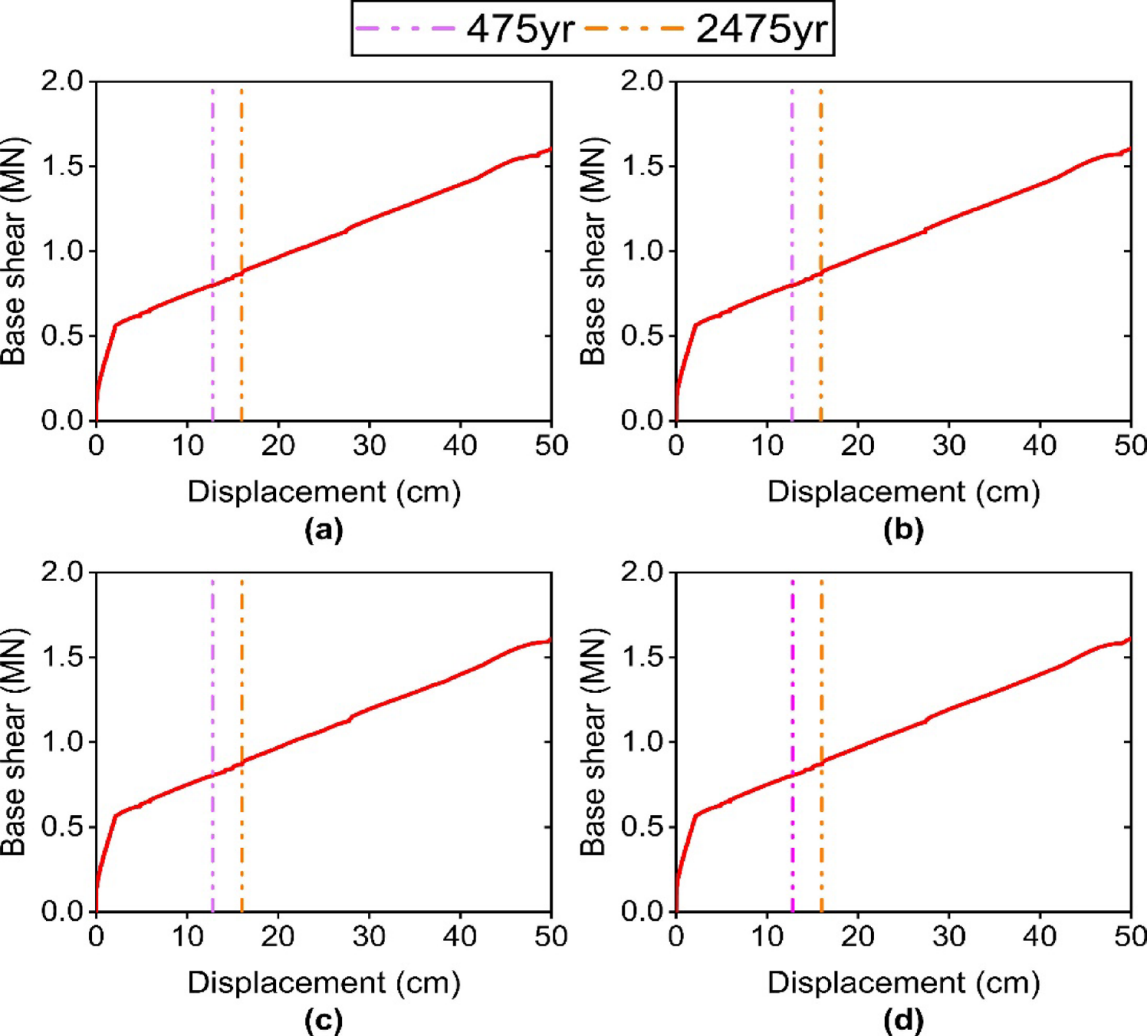
Force-displacement response of TFPI: (**a**) S1 specimen; (**b**) S2 specimen; (**c**) S3 specimen; (**d**) S4 specimen.

Figure 11 shows the force-displacement hysteresis curves of the isolator for the structure with different Satchel connections. As can be observed, the change in connection stiffness has a negligible effect on the hysteretic behavior of the isolator. This indicates that the hysteresis behavior of the isolator is independent of the Satchel connection stiffness. Moreover, these curves show that the displacement capacity of the isolator adequately meets the seismic demand of the structure, and no pounding phenomenon has occurred. Since pounding strongly affects the performance of the isolator, designing the isolator displacement capacity to exceed the minimum recommended by the code can keep the structure safe under earthquakes with intensities beyond the level prescribed in the codes.

**Fig. 12:**
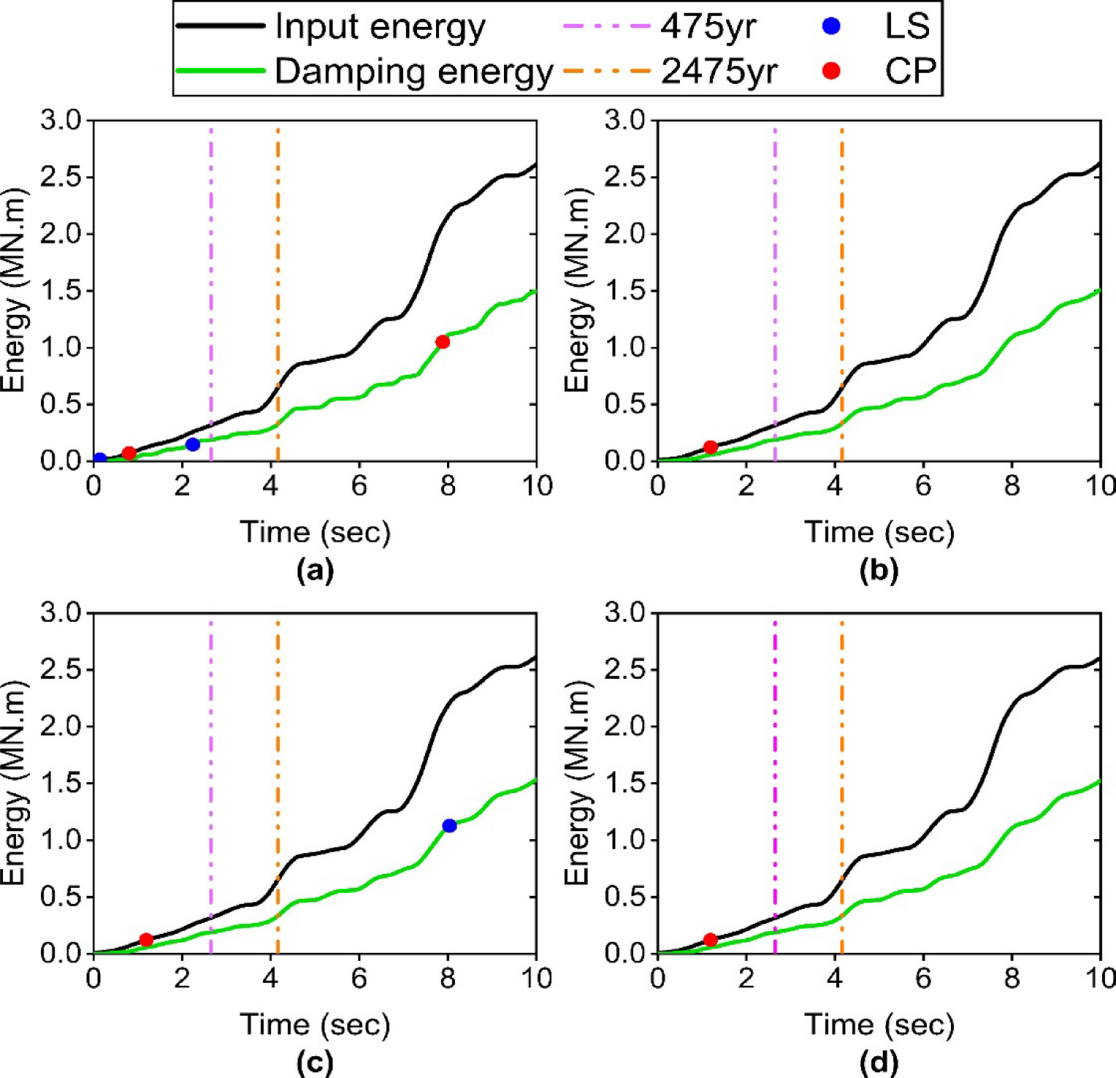
Input energy and damping energy by TFPI: (**a**) S1 specimen; (**b**) S2 specimen; (**c**) S3 specimen; (**d**) S4 specimen.

This is also evident in Fig. 12, which demonstrates that the isolator’s performance in dissipating input energy is independent of the Satchel connection stiffness. Specifically, under the 475-year return period earthquake, the percentages of input energy dissipated by the isolator for specimens S1 to S4 are 59.37%, 60.12%, 60.11%, and 60.25%, respectively, values that are notably similar. Moreover, the corresponding values are 50.33%, 51.43%, 51.40%, and 51.45% under the earthquake with a 2475-year return period.

## Verification

**Fig. 13:**
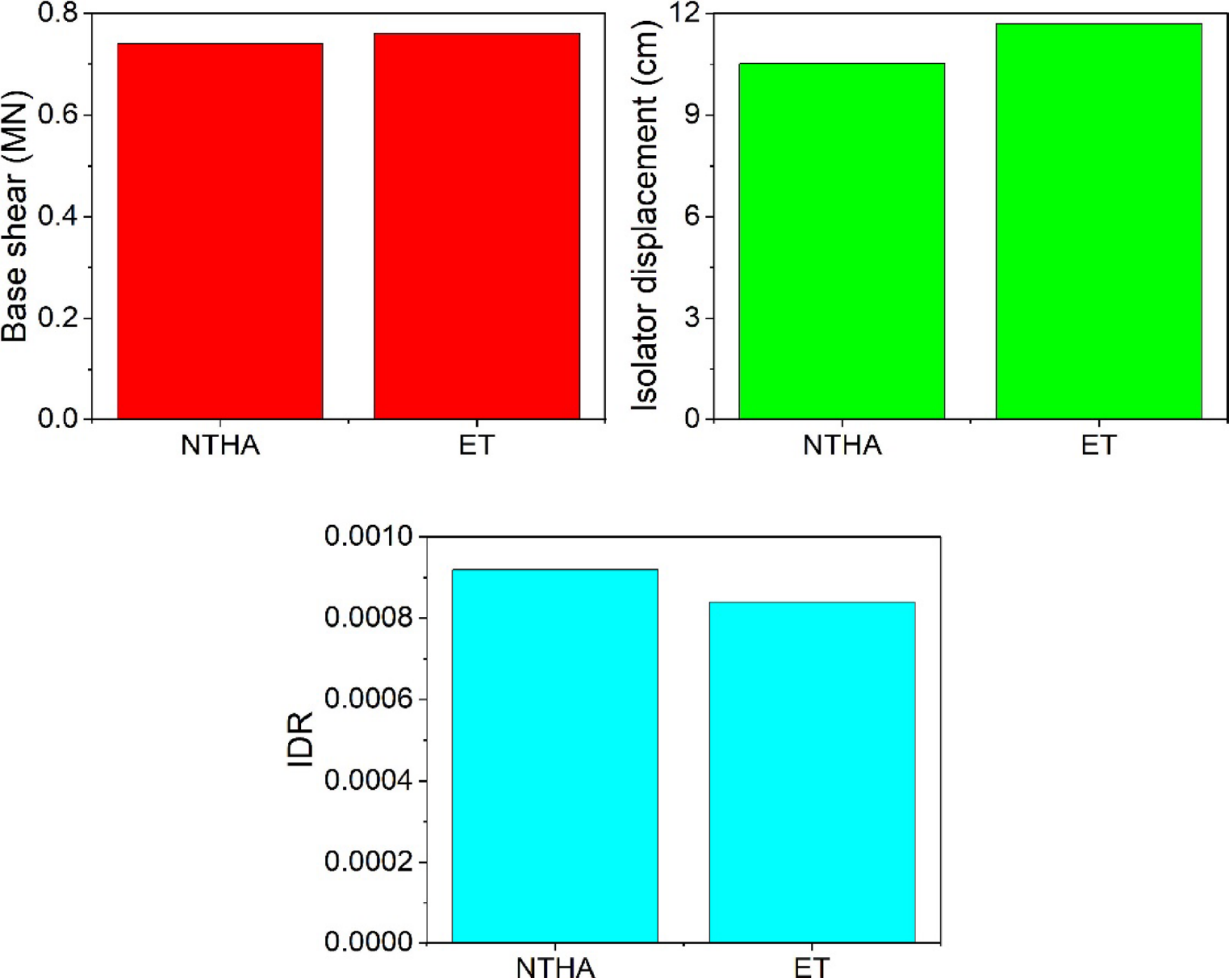
Comparison of ET and NTHA results: (**a**) Base shear; (**b**) Isolator displacement; (**c**) IDR.

In this section, to verify the accuracy of the results obtained from the ET method, specimen S1 is analyzed under the 22 far-field earthquake records proposed by FEMA P-695^28^ (Table 3). The ET excitation functions (ETEFs) used in this study are derived from these records. The results from the ET analysis and the nonlinear time history analysis (NTHA) under the 475-year return-period earthquake are presented in Fig. 13. The average values from the NTHA for the maximum base shear, IDR, and isolator displacement are 0.74MN, 0.00091, and 10.52 cm, respectively. The corresponding values from the ET analysis are 0.76MN, 0.00084, and 11.7 cm. The comparison shows an acceptable level of agreement between the two methods, confirming the reliability of the ET method results.

**Table 3:** Properties of far-field ground motions.

Event	Station	Component	Year	Mag.	Vs30 (m/sec)	PGA (g)
San Fernando	LA - Hollywood Stor	PEL090	1971	6.6	316	0.21
Friuli	Tolmezzo	A-TMZ000	1976	6.5	425	0.35
Imperial Valley	El Centro Array #11	H-E11140	1979	6.5	196	0.36
Imperial Valley	Delta	H-DLT262	1979	6.5	275	0.24
Superstition Hills	Poe Road (temp)	B-POE270	1987	6.5	208	0.45
Superstition Hills	El Centro Imp. Co.	B-ICC000	1987	6.5	192	0.36
Loma Prieta	Gilroy Array #3	G03000	1989	6.9	350	0.56
Loma Prieta	Capitola	CAP000	1989	6.9	289	0.53
Manjil	Abbar	ABBAR-L	1990	7.4	724	0.51
Landers	Coolwater	CLW-LN	1992	7.3	271	0.28
Cape Mendocino	Rio Dell Overpass	RIO270	1992	7.0	312	0.39
Landers	Yermo Fire Station	YER270	1992	7.3	354	0.24
Northridge	Canyon Country-WLC	LOS000	1994	6.7	309	0.41
Northridge	Beverly Hills - Mulhol	MUL009	1994	6.7	356	0.42
Kobe	Nishi-Akashi	NIS000	1995	6.9	609	0.51
Kobe	Shin-Osaka	SHI000	1995	6.9	256	0.24
Kocaeli	Arcelik	ARC000	1999	7.5	523	0.22
Hector Mine	Hector	HEC000	1999	7.1	685	0.27
Duzce	Bolu	BOL000	1999	7.1	326	0.73
Chi-Chi	TCU045	TCU045-E	1999	7.6	705	0.47
Kocaeli	Duzce	DZC180	1999	7.5	276	0.31
Chi-Chi	CHY101	CHY101-E	1999	7.6	259	0.35

## Conclusions

This research aimed to determine the effectiveness of seismic isolation as a retrofit measure to improve the seismic performance of steel structures equipped with Satchel connections, which have historically demonstrated a susceptibility to premature weld failure. To this end, a five-story steel building with four different Satchel connection properties (S1 to S4) was modeled under both fixed-base and base-isolated conditions. The structural seismic response was analyzed using the nonlinear endurance time analysis method under various seismic hazard levels. The assessment focused on two critical performance parameters: structural elements and acceleration-sensitive non-structural components. The key conclusions of this study are as follows:

1. The seismic isolation system significantly reduced the interstory drift ratio (IDR) by approximately 7 times at the 475-year hazard level and by about 5.5 to 7 times at the 2475-year hazard level. Moreover, the isolated structural elements remained entirely within the Immediate Occupancy (IO) performance level, even under very severe earthquakes exceeding the 2475-year return period. Most isolated specimens either showed no plastic hinge formation or limited their behavior to the Life Safety (LS) level. Conversely, the fixed-base structure performed poorly, reaching the Collapse Prevention (CP) level before the 475-year hazard level.
2. The absolute floor acceleration, the primary parameter influencing acceleration-sensitive non-structural components, was substantially mitigated by employing isolators. This reduction was approximately 7-fold for the 475-year return period earthquake and over 6-fold for the 2475-year return period. Therefore, the predicted damage to non-structural elements was downgraded from extensive in the fixed-base structure to none to slight in the isolated structure. This benefit is especially valuable for essential facilities like hospitals and critical buildings, where maintaining the functionality of equipment is paramount.
3. A substantial reduction was achieved in the base shear, the critical design force for the foundation and isolation system. At the 2475-year return period, this force diminished from approximately 4.5MN to below 0.95MN, representing a decrease by a factor exceeding 4.7. This significant mitigation of the lateral force permits the implementation of more cost-effective isolators and facilitates a marked reduction in foundation retrofitting expenditure.
4. Increasing the stiffness of the Satchel connections (from specimen S1 to S4, achieved by increasing the angle length and enlarging the beam and angle sections) showed little to no effect on the structural response when base isolators were present. In some instances, it resulted in only a marginal increase. This indicates that extensive strengthening of Satchel connections is not required when a base isolation system is implemented. Performance significantly exceeding that of a new, code-compliant design can be achieved simply by adding isolators to the existing connections.

In this study, a structure that is regular in both plan and elevation is used. Therefore, the influence of torsion on the rehabilitated structure’s behavior remains an area for future research. Moreover, structural performance under near-fault ground motions was not examined. Due to their unique characteristics, particularly the presence of strong velocity pulses, investigating this aspect is recommended for future work.

## Data Availability

The datasets generated during and/or analysed during the current study are available from the corresponding author on reasonable request.

## References

[1] Grubits, P. et al. Structural topology optimization for plastic-limit behavior of I-beams, considering various beam-column connections. *Mech. Based Des. Struct. Mach.* **53** (4), 2719–2743 (2025).

[2] Lógó, J., Movahedi Rad, M., Knabel, J. & Tauzowski, P. Reliability based design of frames with limited residual strain energy capacity. *Periodica Polytech. Civil Eng.* **55** (1), 13–20 (2011).

[3] Shafei, B., Mirghaderi, S., Motavalli, M. & Lestuzzi, P. Seismic evaluation and upgrading of typical iranian steel buildings. In *The 14th World Conference on Earthquake Engineering* (2008).

[4] Tehranizadeh, M. Approximate parameter for semi-rigid ‘Khorjinee’ connections in dynamic torsional response of steel structures. *Eng. Struct.* **22**, 335–342 (2000).

[5] Arbabi, F. Nonlinear deformation of satchel connections. *J. Seismology Earthq. Eng.* **1** (1), 51–57 (1998).

[6] Daryan, A. S. & Bahrampoor, H. Behavior of Khorjini connections in fire. *Fire Saf. J.* **44**, 659–664 (2009).

[7] Mostafaei, H. & Mazroi, A. Experimental study and post-earthquake damage inspection of scissors-type or Satchel (Khorjini) connections for steel-frame buildings. In *13th World Conference on Earthquake Engineering* (2004).

[8] Tehranizadeh, M. Passive energy dissipation device for typical steel frame building in Iran. *Eng. Struct.* **23**, 643–655 (2001).

[9] Mirghaderi, S. & Dehghani Renani, M. The rigid seismic connection of continuous beams to column. *J. Constr. Steel Res.* **64**, 1516–1529 (2008).

[10] Hosseini Hashemi, B. & Hassanzadeh, M. Study of a semi-rigid steel braced building damaged in the Bam earthquake. *J. Constr. Steel Res.* **64**, 704–721 (2008).

[11] FEMA-356. *Prestandard and Commentary for the Seismic Rehabilitation of Buildings* (Building Seismic Safety Council, 2000).

[12] Amiri Hormozaki, H. & Aghakouchak, A. Experimental study of cyclic behavior of conventional saddle kike connections and their acceptance criteria. *Struct. Steel*. **7** (9), 79–96 (2011).

[13] Computers and Structures, Inc. (CSI). SAP2000 Ultimate V. 24.1.0 Build 2035, Analysis Reference Manual for SAP2000, (CSI, 2022).

[14] Ghodrati Amiri, G., Behnamfar, F. & Azad, H. Quantities derivation of thresholds of different seismic performance levels for satchel frames using pushover analysis. *Struct. Steel*. **4** (2), 71–86 (2008).

[15] Sadeghi-Movahhed, A., De Domenico, D. & Majdi, A. Structural flexibility impact on pounding severity and seismic performance of adjacent isolated buildings. *Soil Dyn. Earthq. Eng.* **181**, 108667 (2024).

[16] Sadeghi-Movahhed, A., De Domenico, D., Mashayekhi, M. & Majdi, A. Optimal damping of isolated tall buildings accounting for structural and nonstructural damage. *J. Building Eng.* **105**, 112497 (2025).

[17] Sadeghi-Movahhed, A., Billah, A. H. M. M., Shirkhani, A., Mashayekhi, M. & Majdi, A. Vulnerability assessment of tall isolated steel building under variable earthquake hazard levels using endurance time method. *Journal Struct. Integr. Maintenance* **9** 1 (2024).

[18] Majdi, A., Mashayekhi, M. & Sadeghi-Movahhed, A. Effect of near-fault earthquake characteristics on seismic response of mid-rise structures with triple friction pendulum isolator. *J. Rehabilitation Civil Eng.* **12** (1), 47–62 (2024).

[19] Majdi, A. et al. and M. Movahedi Rad, On critical pounding mechanism of base-isolated buildings using an optimized multi-hazard method. *Results Eng.* **27** 106533 (2025).

[20] Constantinou, M., Kalpakidis, I., Filiatrault, A. & Ecker Lay, R. LRFD-Based analysis and design procedures for bridge bearings and seismic isolators (Technical Report No. MCEER-11-0004), State University of New York at Buffalo, (2011).

[21] Sarlis, A. A. S. & Constantinou, M. C. Modeling triple friction pendulum isolators in program SAP2000. *Document Distributed to the Engineering Community Together with Example*. 27 (2010).

[22] Gudainiyan, J. & Gupta, P. K. Effect of frequency content parameter of ground motion on the response of C-shaped base-isolated building. *Asian J. Civil Eng.* **24**, 2973–2983 (2023).

[23] Sadeghi Movahhed, A., Shirkhani, A., Zardari, S., Noroozinejad Farsangi, E. & Karimi Pour, A. Effective range of base isolation design parameters to improve structural performance under far and near-fault earthquakes. *Adv. Struct. Eng.* **26** (1), 52–71 (2023).

[24] Majdi, A. et al. On the influence of unexpected earthquake severity and dampers placement on isolated structures subjected to pounding using the modified endurance time method. *Buildings.* **13**(5) 1278 (2023).

[25] Sadeghi Movahhed, A. et al. Modified endurance time method for seismic performance assessment of base-isolated structures. *J. Building Eng.* **67**, 105955 (2023).

[26] Majdi, A. et al. Application of the modified endurance time method for predicting seismic response of base-isolated structures under pounding. *J. Earthq. Tsunami*. **19** (3), 2450037 (2025).

[27] Estekanchi, H. E. et al. A state-of-knowledge review on the endurance time method, *Structures.* **27** 2288–2299 (2020).

[28] FEMA P-695. *Quantification of Building Seismic Performance Factors*. (2009).

[29] Mashayekhi, M., Estekanchi, H. E., Vafai, H. & Mirfarhadi, S. A. Development of hysteretic energy compatible endurance time excitations and its application. *Engineering Structures.* **177** 753–769 (2018).

[30] ASCE41-06. *Seismic Rehabilitation of Existing Buildings* (American Society of Civil Engineers, 2007).

[31] Hazus 4.2. *Hazus Earthquake Model Technical Manual* (Federal Emergency Management Agency, 2020).

